# Homozygous mutation of focal adhesion kinase in embryonic stem cell derived neurons: normal electrophysiological and morphological properties *in vitro*

**DOI:** 10.1186/1471-2202-7-47

**Published:** 2006-06-12

**Authors:** P Charlesworth, NH Komiyama, SGN Grant

**Affiliations:** 1 Wellcome Trust Sanger Institute, Hinxton, Cambridge, CB10 1SA, UK; 2 Centre for Neuroscience Research, University of Edinburgh, Edinburgh, UK

## Abstract

**Background:**

Genetically manipulated embryonic stem (ES) cell derived neurons (ESNs) provide a powerful system with which to study the consequences of gene manipulation in mature, synaptically connected neurons *in vitro*. Here we report a study of focal adhesion kinase (FAK), which has been implicated in synapse formation and regulation of ion channels, using the ESN system to circumvent the embryonic lethality of homozygous FAK mutant mice.

**Results:**

Mouse ES cells carrying homozygous null mutations (FAK^-/-^) were generated and differentiated *in vitro *into neurons. FAK^-/- ^ESNs extended axons and dendrites and formed morphologically and electrophysiologically intact synapses. A detailed study of NMDA receptor gated currents and voltage sensitive calcium currents revealed no difference in their magnitude, or modulation by tyrosine kinases.

**Conclusion:**

FAK does not have an obligatory role in neuronal differentiation, synapse formation or the expression of NMDA receptor or voltage-gated calcium currents under the conditions used in this study. The use of genetically modified ESNs has great potential for rapidly and effectively examining the consequences of neuronal gene manipulation and is complementary to mouse studies.

## Background

A major goal in post-genomic neuroscience is to elucidate the function of individual genes in neurons. Gene targeting is one of the most widely used methods to study the function of genes in the vertebrate nervous system and in recent years the need for conditional mutation strategies has been emphasised. For example, the tetracycline system has been used to transiently express genes in the brain [1] and the Cre/*LoxP *system used to delete genes in selected neuronal populations [2]. One disadvantage to these techniques is that it can take 2 or more years for results, in part because of the breeding of mice. Therefore any method that allows for a shorter duration and reduces the need for animals is potentially useful. Toward this end, we have adopted an experimental strategy using genetically engineered embryonic stem (ES) cell derived neurons (ESNs). This system has the potential to greatly speed the functional analysis of genetic manipulations and importantly also allows access to lethal mouse phenotypes.

**Figure 1:**
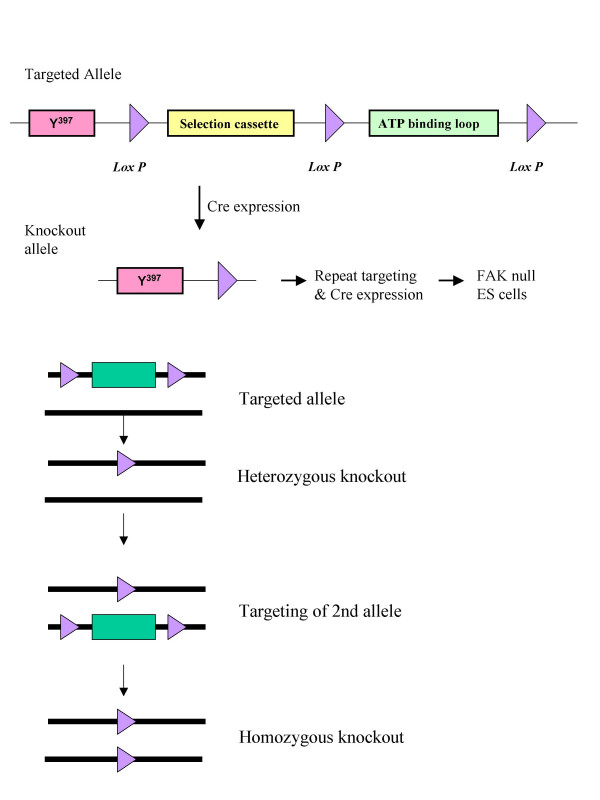
**FAK locus targeting**. Simplified diagram of FAK locus targeting strategy. The top of the panel shows the targeted allele containing 3 loxP sites. Below is shown one of the deletion products following cre recombinase expression, with ATP binding loops deleted. This was used in the present study to generate homozygous FAK null ES cells after a second round of targeting and deletion, depicted schematically in the lower panel. For further details see additional file [1]1and [9].

Totipotent murine ES cells can be induced to differentiate *in vitro *to form mature post-mitotic neurons, which in addition to being synaptically connected, show many properties comparable to primary cultures of embryonic neonatal cortex [3-5]. The use of genetically manipulated ES cells to study knockout of gene function in neurons has however thus far not been extensively exploited [6-8]. In these studies, the spontaneous conversion of a heterozygous to a homozygous knockout ES cell has been selected using high concentrations of neomycin. An alternative and more reliable strategy, used in this study, involves the use of Cre/*loxP *to create the homozygous mutation in sequential rounds of gene targeting (see Fig. 1, and additional file 1). This has particular advantages, allowing precise targeting of both alleles using the same vector and the ES cell line carrying mutations in which a gene was flanked by two loxP sites may be used to generate a conditional, tissue specific, knock-out mouse (see [9]).

Genetically modified mice have become a standard tool to examine the role of synaptic proteins in the biology of neurotransmission, synaptic plasticity and behaviour. The first studies of tyrosine kinases in synaptic plasticity [10,11] and learning [10] revealed an important role for fyn tyrosine kinase. The lack of Fyn resulted in reduction in glutamate receptor signaling and the impaired induction of long-term potentiation. In addition, there were observed changes in the morphology of the dendritic tree of hippocampus pyramidal neurons and learning deficits. Since Fyn was a Src family kinase, subsequent studies on known Src substrates lead to the finding that FAK was hypophosphorylated in *fyn *mutant mice and this was associated with reduced kinase activity [12]. Moreover, studies in non-neuronal cells showed that FAK was responsible for phosphorylation of several proteins involved in cell-cell contacts (see below) and thus a likely mediator of the synaptic phenotype of *fyn *mutant mice. In addition, Rico and colleagues showed that *in vivo *the deletion of FAK resulted in abnormal architecuture of neurons [13]. Thus FAK may play an important role in the formation and function of synapses and neurotransmission.

Unfortunately, mice lacking FAK die in early embryogenesis before neurons can be cultured (Mice carrying homozygous FAK null mutation die at an early stage in embryonic development (e 8.5) [15], although use has been made recently of conditional FAK knockout mice [13,16,17] to circumvent this problem. An alternative approach is the creation of neurons derived directly from homozygous mutant embryonic stem cells.

FAK is a 125 KDa non-receptor protein-tyrosine kinase localised at focal adhesion plaques where cells attach to the extracellular matrix and much work has focused on the role of FAK in cell adhesion, specifically as a key mediator of signalling events initiated by integrin binding and activation of FAK by autophosphorylation [18]. FAK possesses binding sites for a number of proteins, most importantly, Src family kinases [19]. These link FAK to a variety of signaling pathways, including the MAPK pathway. FAK is highly expressed in the brain and is activated by a variety of stimuli, such as neurotransmitters (including glutamate and acetylcholine), lipid messengers (such as anandamide, arachidonic acid and lysophatidic acid), growth factors (insulin, NGF, endothelin) and NCAM [20,21]). The identification of neuronal and brain specific splice isoforms of FAK generated by alternative RNA splicing [22] also suggested that FAK may have roles in neurons beyond, or independent of, cell attachment. Furthermore, unlike the restricted localisation of FAK to focal contacts in non-neuronal cells, FAK is expressed throughout neurons, suggesting FAK has different mechanisms for subcellular localisation in neurons, and, potentially, has novel targets [23]. Since neuronal FAK appears to have functions other than adhesion/motility in neurons, we explored the possibilities, principally using the whole cell patch clamp technique, that FAK is instead involved in ion channel modulation and synapse formation. We focused our attention on two channel types with pivotal roles in neuronal signaling pathways: the NMDA receptor and voltage-gated calcium channels.

## Results

### FAK expression in wild type and mutant cells

**Figure 2:**
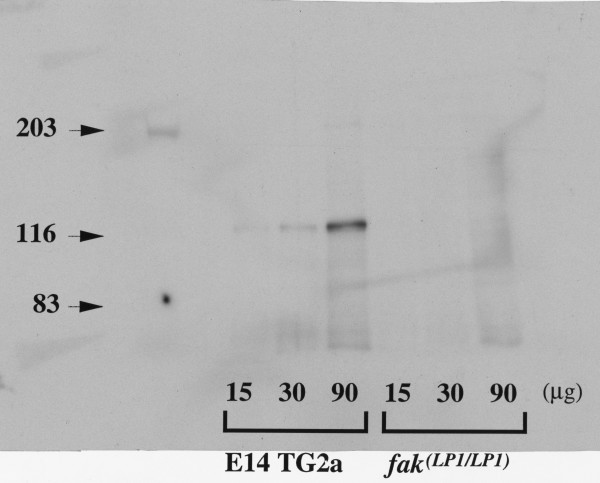
Western blot of cellular extract of wild type and FAK null ES cells.

Western blot analyses using FAK monoclonal antibody were performed on cellular extracts to examine FAK expression in wild type (WT) and FAK null ES cells. Results showed a single band whose molecular weight was approximately 125 KDa in E14TG2a lines. No signal was detected in FAK null homozygous lines (see Fig. 2). Additionally, McLean et al [9] found no expression of a truncated product using N-terminal FAK antibodies. PCR analysis of RNA extracted from WT ESNs (10 days *in vitro*) was performed using primers hybridising either side of the FAK neuronal specific exons [22]. The resulting product contained bands corresponding to neuron-specific splice isoforms of FAK, in addition to non-neuronal spliced FAK, the latter presumably due to the presence of non-neuronal cells in the differentiated ES cell culture.

### FAK null ESNs display normal growth and morphology

After plating the cell suspension created by enzymatic dispersal of the trans-retinoic acid (RA) treated embryoid bodies, cells rapidly adhered to the substrate (laminin). In both wild type and FAK -/- cultures cells with rudimentary processes were evident within 24–48 hrs. With increasing time in culture, cells with progressively longer processes and more complex branching were observed. Again, no clear difference was observed in the length or complexity of processes between wild type and FAK -/- cells at comparable culture times. As evidenced by the ability to record synaptic currents (see below) synapse formation began at approx. 5–7 days *in vitro *(DIV), and progressed thereafter. An approximate quantitation of cell growth was derived by measuring the apparent cell capacitance in whole cell patch clamp mode, which was done at the start of each recording, and plotting this value against DIV. Whole cell capacitance increased by 1.22 ± 0.06 pF/day (n = 85) in FAK -/- cells, and by 1.15 ± 0.05 pF/day (n = 119) in wild type cells.

**Figure 3:**
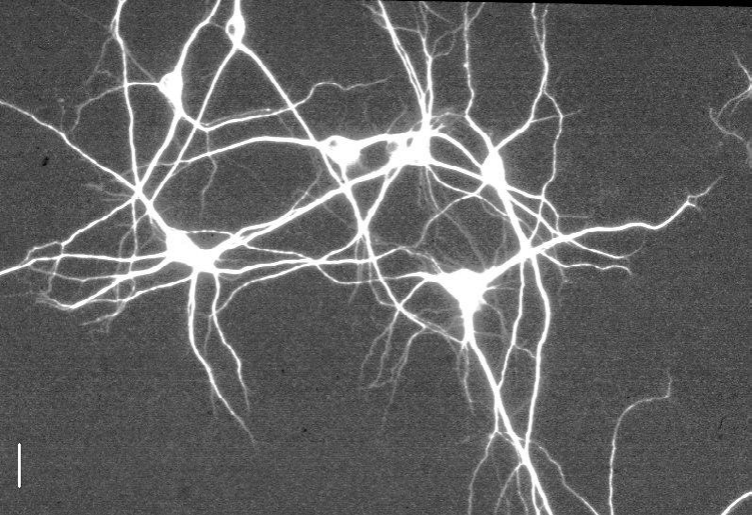
**Immunohistochemistry of wild type and FAK null ESNs**. WT ESN 15 DIV. MAP2B ×20. **Scale bars**: in fig. 3: 50 μm; in figs 5 & 7: 10 μm.

**Figure 4:**
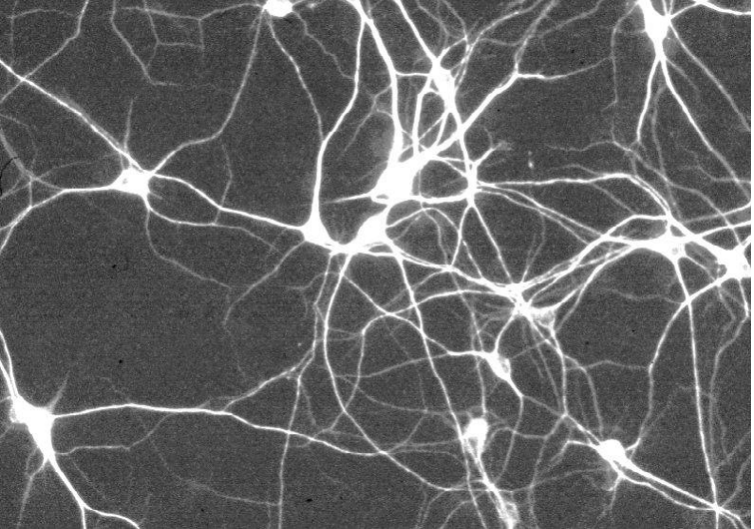
**Immunohistochemistry of wild type and FAK null ESNs**. FAK -/- ESN 15 DIV. MAP2B ×20. **Scale bars**: in fig. 3: 50 μm; in figs 5 & 7: 10 μm.

**Figure 5:**
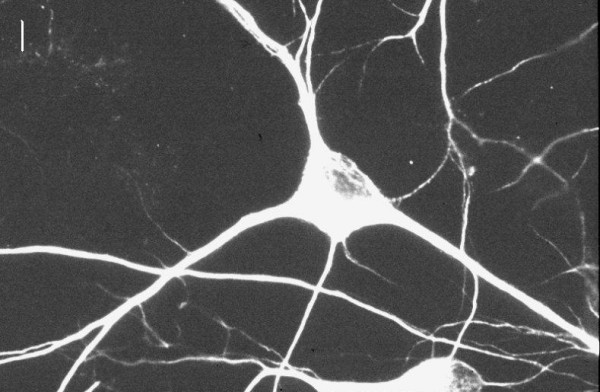
**Immunohistochemistry of wild type and FAK null ESNs**. WT ESN 15 DIV. MAP2B ×100. **Scale bars**: in fig. 3: 50 μm; in figs 5 & 7: 10 μm.

**Figure 6:**
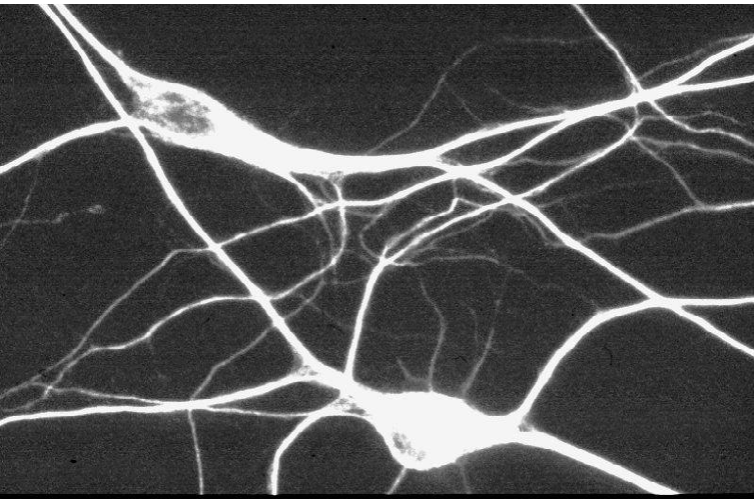
**Immunohistochemistry of wild type and FAK null ESNs**. FAK -/- ESN 15 DIV. MAP2B ×100. **Scale bars**: in fig. 3: 50 μm; in figs 5 & 7: 10 μm.

**Figure 7:**
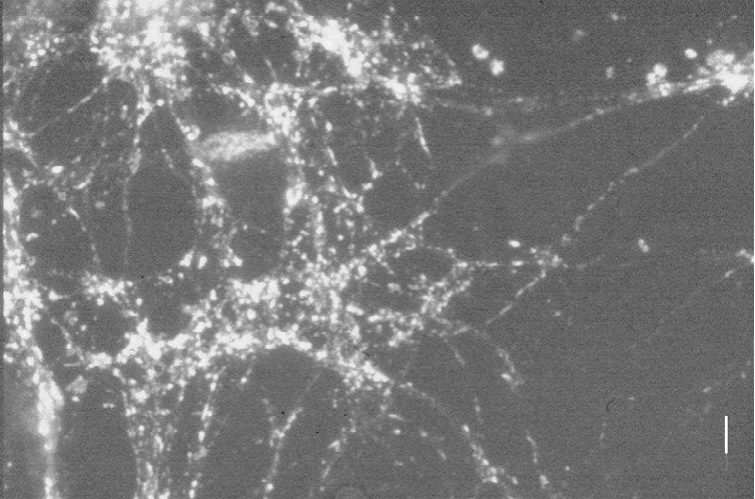
**Immunohistochemistry of wild type and FAK null ESNs**. WT ESN 15 DIV. synaptophysin ×100. **Scale bars**: in fig. 3: 50 μm; in figs 5 & 7: 10 μm.

**Figure 8:**
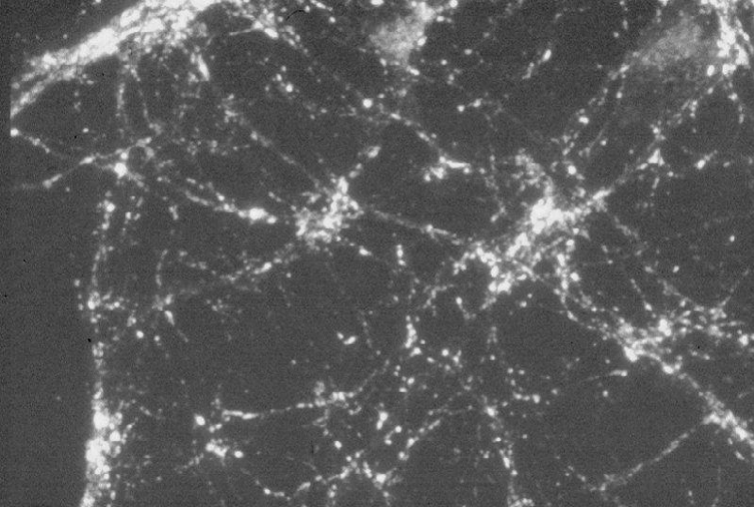
**Immunohistochemistry of wild type and FAK null ESNs**. FAK -/- ESN 15 DIV. synaptophysin ×100. **Scale bars**: in fig. 3: 50 μm; in figs 5 & 7: 10 μm.

Immunohistochemistry was performed on ESN cultures (8–15 DIV) using monoclonal antibodies to the dendritic protein MAP2B and the pre-synaptic protein synaptophysin, staining being visualised with Cy3 conjugated secondary antibody. The results showed (see Fig 3, 4, 5, 6, 7, 8) the extent and complexity of neuronal processes in both wild type and FAK -/- ESN cultures, and the extensive formation of synapses in both.

### Electrophysiological properties of wild type and Fak null ESNs

Having established that FAK +/+ and FAK -/- ESNs differentiated from ES cells in a similar manner, we next asked if key electrophysiological parameters were also comparable in these cells, and to the properties expected of primary cultured cortical neurons consistent with previous reports [3-5].

Cells were selected for whole cell patch clamp recording on the basis of a neuronal morphology similar to that of primary cultured cortical neurons. Most cells recorded from had a pyramidal morphology (see e.g fig. 5) and ranged in age from 3 to 21 days in culture.

**Figure 9:**
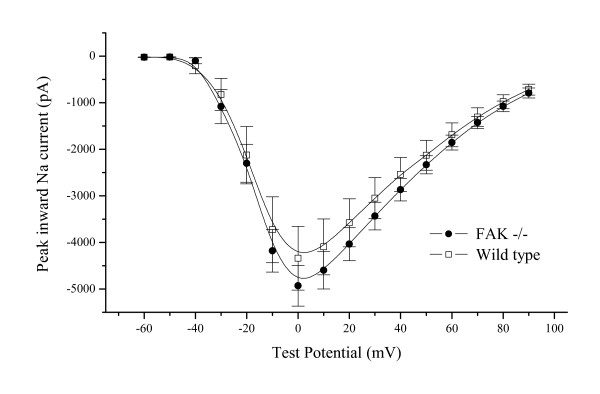
**Voltage-gated sodium and calcium currents in wild type and FAK null ESNs**. Current – voltage relationships for TTX-sensitive voltage-gated sodium currents in wild type (n = 24) and FAK null (n = 27) ESNs.

**Figure 10:**
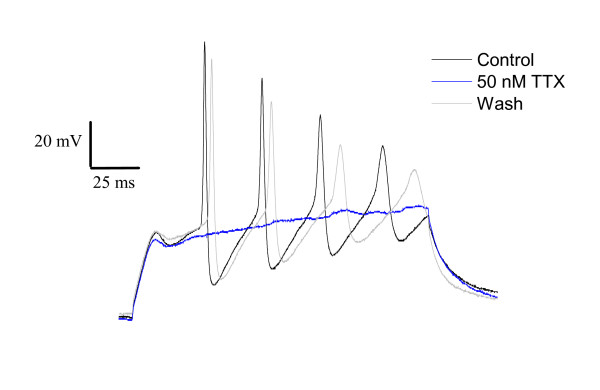
**Voltage-gated sodium and calcium currents in wild type and FAK null ESNs**. Action potentials in a wild type ESN elicited by depolarising current injection (0.15 nA, 160 ms), showing reversible block by 50 nM TTX. (TTX trace recorded 1 min after application, wash recorded 3 min after return to control).

Rapidly activating, rapidly inactivating inward currents were evoked by short depolarising voltage pulses in standard bathing medium. All cells tested displayed such currents, though at the earliest time points (2–3 d post-plating) they were very small. This fast inward current was completely blocked by 50–200 nM TTX. There was no obvious difference in this TTX-sensitive Na current between FAK -/- and wild type ESNs in respect of magnitude, kinetics, and IV relationship. Rapidly inactivating TTX- sensitive currents were activated at potentials positive to -40 mV, with peak current elicited by test potentials of ~0 mV (fig 9). Peak I_Na _was 5145 pA ± 496 n = 25 in FAK -/- ESN and 4671 pA ± 861 n = 18 in wild type ESNs. As expected from the robust expression of this current, when cells were current-clamped and sufficient depolarising current was injected, trains of fast action potentials were evoked. These were reversibly blocked by 50 nM TTX (see fig 10).

**Figure 11:**
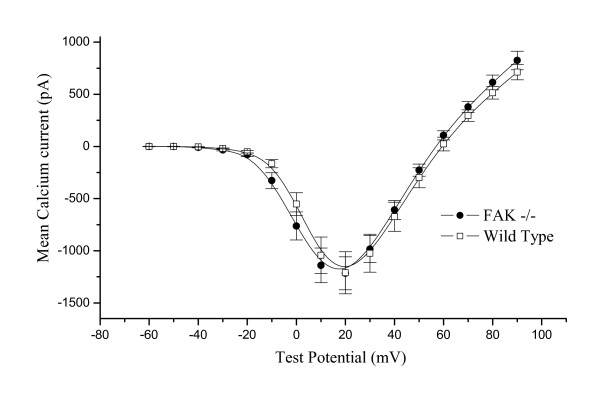
**Voltage-gated sodium and calcium currents in wild type and FAK null ESNs**. Current – voltage relationships for voltage-gated calcium currents in wild type (n = 23) and FAK null (n = 30) ESNs. Currents were evoked by 20 ms step command potentials from a holding potential of -70 mV. No significant differences at any voltage.

Voltage-gated calcium currents were isolated using the solutions described in the methods and recorded in the whole cell patch clamp configuration (fig. 11). The current – voltage relationship was determined by applying step command potentials (20 ms duration) ranging from -60 to +90 mV. For both FAK -/- and wild type ESNs this activation curve was typical of neuronal high voltage activated Ca current: currents were evoked by steps positive to -30 mV, peak currents elicited at +20 mV, with reversal at ~+60 mV. The magnitude of peak calcium current was not significantly different in FAK -/- and wild type ESNs: 1166 ± 189 pA, n = 25 for WT; 1393 ± 172 n = 32 for FAK null. Expressed as current density (peak current/cell capacitance), corresponding values were 69.2 ± 5.8 pA/pF for WT and 68.9 ± 6.8 pA/pF for FAK null neurons. Additionally, the time constant of calcium current activation following a depolarising voltage pulse was not significantly different in FAK -/- and wild type ESNs. For steps to +20 mV, τ were 1.39 ± 0.13 ms in wild type and 1.20 ± 0.13 ms in FAK null ESNs.

The specific N-type calcium channel blocker, ω-conotoxin GVIA (2 μM) irreversibly inhibited a proportion of the high voltage activated calcium current in both FAK -/- and wild type ESNs (WT 40.4 ± 4.8% inhibition n = 5; FAK- 49.1 ± 4.6% n = 6). While the inhibition was somewhat greater in the FAK null cells, the difference was not statistically significant. ω-conotoxin MVIIC (2 μM) inhibited currents in a partly reversible manner, consistent with its reported action as an (almost) irreversible inhibitor of P/Q type channels, but a reversible inhibitor of N-type. In one WT ESN acute blockade was 43.0%, while the sustained level of inhibition (6 mins after toxin) was 22.0%. Corresponding figures for a FAK – ESN were 42.5 and 20.8%.

**Figure 12:**
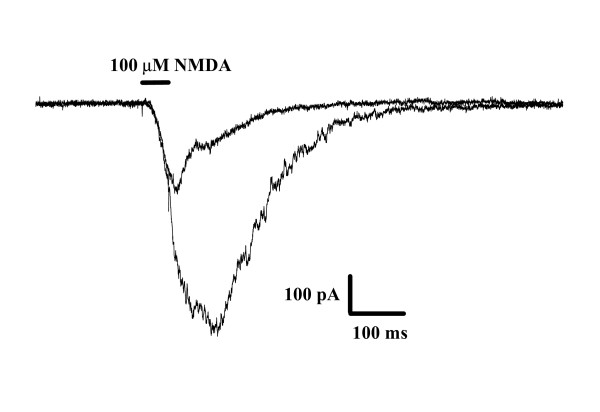
**NMDA currents in wild type and FAK null ESNs**. Example of NMDA current recorded in the presence and absence of 1 mM Mg in a FAK null ESN.

Currents gated by the NMDA subtype of glutamate receptor were evoked by brief application (see methods) of 100 μM NMDA/10 μM glycine and recorded in the whole cell patch clamp configuration. For these experiments, nominally magnesium free bathing solution was used. Peak NMDA currents (recorded at a holding potential of -70 mV) were not significantly different in the WT and FAK null ESNs: WT 1253 ± 177 pA; FAK -/- 1387 ± 137 pA. In magnesium free solutions, the current – voltage relationship of NMDA receptor gated currents was linear, while in the presence of 1 mM Mg^2+ ^a marked attenuation of the current was observed at hyperpolarised potentials (fig 12). This voltage-dependent Mg^2+ ^block was seen in both WT and FAK null ESNs.

Spontaneous miniature synaptic currents (mEPSCs) were recorded (in the presence of TTX) in cells from ~7 d in culture, strongly suggesting the formation of functional synapses, in both WT and FAK null ESN cultures. On the basis of their kinetics, these mEPSCs were glutamatergic in origin.

### Effect of genistein on NMDA and voltage-gated calcium currents

A number of studies have shown that Src-family tyrosine kinases modulate the activity of both voltage-gated calcium currents [24] and NMDA receptors [25,26]. Since FAK is known to be involved with Src-family kinase function, we tested the effect of inhibiting tyrosine kinase activity in wild type compared to FAK null ESNs using the specific inhibitor genistein (4',5,7 trihydroxyisoflavone), and the related inactive compound, daidzein (4',7 dihydroxyisoflavone).

Calcium currents were evoked by 40 ms step depolarisations from -70 mV to +10 mV delivered at 0.1 Hz. After a control period of 8–10 stimuli 50 μM genistein was applied via bath perfusion for 120 s. In both FAK -/- and wild type ESNs calcium currents were reversibly inhibited by genistein, and to a similar degree: WT 34.9% inhibition ± 1.0 n = 9; FAK -/- 33.8% ± 0.8 n = 10 similar to results obtained by Potier & Rovira [24] This inhibition was relatively rapid with 80% of maximal inhibition achieved within 60 s. Daidzein (50 μM) also inhibited currents, though to a lesser extent: WT 17.1% inhibition ± 2.2 n = 5; FAK -/- 13.1% ± 1.3 n = 6. This may suggest that a component of the genistein effect was not related to tyrosine kinase inhibition, but may have been due to a direct action on calcium channels [27].

**Figure 13:**
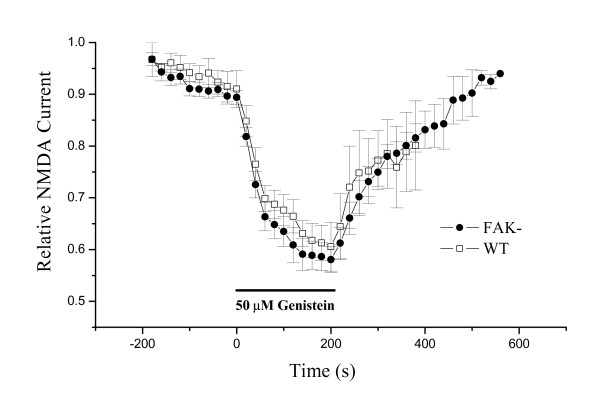
**NMDA currents in wild type and FAK null ESNs**. Time course of NMDA current inhibition by genistein. Currents were evoked every 20 s by 100 ms application of 100 μM NMDA. 50 μM genistein was bath applied for 4 minutes.

NMDA receptor gated currents were also reversibly inhibited by 2 minute bath application of genistein (50 μM). Again, a similar degree of inhibition was seen in both WT and FAK null ESNs: WT 35.4 ± 2.7% inhibition n = 6; FAK -/- 34.6 ± 3.5% n = 12 (fig 13). This was similar to the effect observed by Wang & Salter [28] of a 32% depression in cultured spinal neurons. Daidzein had a minimal effect on NMDA currents in either cell type. In one case (FAK -/- ESN) the slow glutamergic mini component, presumably due to NMDA receptor current, was reversibly inhibited by 50 μM genistein. Thus the effect of genistein on the whole cell NMDA receptor population was replicated at synaptic NMDA receptors.

## Discussion

### The phenotype of FAK null ESNs

Focal adhesion kinase (FAK) null embryonic stem cells (ES) generated by targeting the *fak *locus are able to differentiate into neurones following embryoid body formation and retinoic acid treatment. These neurons were not obviously impaired in their ability to attach to a laminin substrate and extend neurites. Furthermore, they were able to form functional synapses. Earlier studies (reviewed in [18]) had strongly implicated FAK in neuronal adhesion and process outgrowth, though in the absence of a means to specifically and completely ablate FAK it has not previously been possible to test this rigorously. The expression of functional NMDA receptors and voltage-gated calcium channels, and their modulation by tyrosine phosphorylation were also unaltered in the FAK null ESNs. It therefore appears that FAK is not involved in the tonic regulation of these two channel types. In both cases, the level of current inhibition by genistein was similar to that observed by previous studies [24,28]. The functional expression of N-type Ca channels, which are linked to neurotransmitter release, was unaltered in the mutant ESNs.

The absence of a detectable morphological and electrophysiological phenotype in FAK null ESNs raises several possible explanations. Firstly, it is possible that in neurons functions performed by FAK are subsumed by the related tyrosine kinase PYK2 [29] in the FAK null cells. Compensation for certain FAK functions (e.g. integrin activation of MAP kinase) by PYK2 has been observed in FAK null fibroblasts [30,31]. Overlapping and distinct roles for PYK2 and FAK in two neuronal cell lines have been described [32] and also that PYK2 alone is sufficient to enable neurite outgrowth in PC12 cells. This is supported by Park et al [33] who found that PYK2, rather than FAK, was primary involved in NGF-induced neuronal differentiation in PC12 cells. These issues may be addressed in the future with PYK2/FAK double mutant ESNs.

Secondly, the possibility remains that intracellular signalling pathways in ESNs are fundamentally different from those in the neuronal tissues used in previous studies. The available evidence would suggest that this is not the case. For example, β1 integrin null ES cells display severely retarded neuronal differentiation, with severely limited neurite outgrowth [6,34]. Thus far, no important physiological or biochemical differences have been observed between ESNs and primary cultured neurons. It is also possible, albeit unlikely, that during *in vitro *differentiation we are selecting for FAK independent subpopulation of neurons and/or FAK is required in neurons *in vivo *but not in cultured neurons.

Finally, it is of course also possible that FAK is involved in processes beyond the focus of this study. FAK may participate in pathways leading to modulation of other channel types, or (more complex) physiological phenomena, such as apoptosis [35] or synaptic plasticity, [12,14] the latter possibly mediated by binding to SAPAP3 and PSD-95 [36]. In relation to the present study, it is interesting to note that while synaptic plasticity is altered in PSD-95 mutants, NMDA receptor currents are not [37].

Work using mice with a targeted deletion of *fak *in developing forebrain has shown that, *in vivo*, FAK may influence neuronal function indirectly as a result of its expression in non-neuronal cell types resulting in cortical dysplasia due to aberrant neuronal migration[16]. We would not expect to observe such cell autonomous effects of FAK in our ESN system. Using cortical cultures from these conditional knock out mice [17] it was shown that FAK ablation did not alter axon outgrowth *in vitro*, in agreement with our findings. Netrin stimulated attraction was however altered, but this was not examined in our work. Similarly, we did not analyse dendritic spines (which are not visualised by MAP2B staining), and have been shown to be morphologically altered by FAK deletion in an *in vitro *study by Moeller et al [38].

### ESNs as a model system to study neuronal genes

In a more general context, we believe that ES cell derived neurons, with properties comparable to primary cultures of embryonic or neonatal cortex, provide a powerful system in which to study the consequences of neural gene manipulation. There are a number of significant advantages afforded by this methodology. ES cells are readily transfected and appropriate clones selected to yield stable mutant cell lines. Furthermore, since ES cells have a euploid karyotype it is possible to produce precise targeted disruptions of a gene. Both these features afford considerable advantages over strategies involving the transfection of primary cultures, organotypic slices, or transformed cell lines, for example low and variable transfection rate or the existence of endogenous gene product. The study of neuronal gene alterations are now commonly studied in mutant mice, generated from the introduction of engineered ES cells into blastocysts. The production of ESNs by *in vitro *differentiation has the advantage, pertinent to this study, of allowing access to the neuronal phenotype for mutations which give rise to embryonic lethality in mutant mice. In certain cases it may still be possible to obtain mutant neurons by preparing cultures from embryos, however for FAK, lethality is so early as to preclude this and the only cell type which has been derived from the embryos of FAK +/- crosses are fibroblasts [15]. The *in vitro *derivation of ESNs effectively allows the neuronal phenotype of a given mutation to be studied in isolation of more widespread developmental consequences. This technology may of course be used in a complementary manner to mouse studies, since engineered ES cells may be introduced into blastocysts, and, since they can contribute to the germline of chimaeras, mutant mouse lines may be established. Large scale functional studies should now be possible using high throughput gene targeting and trapping techniques and offer unprecedented access to molecular genetic studies of mammalian neurons.

## Conclusion

Focal adhesion kinase (FAK) null embryonic stem cells (ES) may be differentiated in vitro into neurones which extend neurites and form functional synapses. NMDA receptor gated currents and voltage sensitive calcium currents were unaltered in FAK null ESNs, both in the level of their expression and modulation by tyrosine kinases. Embryonic stem cell derived neurons provide a powerful system in which to study the effect of gene manipulation in cells with properties very similar to primary cortical neurones.

## Methods

### Gene targeting and ES cell culture

In order to generate a *fak *null allele as well as a conditional (loxP flanked) allele, a targeting vector carrying three loxP sites in an intronic region of *fak *gene (LP3) was constructed see Fig 1, additional file 1 and [9].

E14 TG2a embryonic stem cells were maintained in feeder free condition on tissue culture plates coated with 0.1% gelatin using the following medium: Glasgow's modified Eagles medium (GMEM) supplemented with 0.1 mM mercaptoethanol, 10% fetal calf serum (FCS) and LIF (100 u/ml).

Not I digested DNA (150 μg) was electroporated into ES cells (1 × 10^8 ^cells) using a Bio-Rad Gene Pulser (0.8 kV, 3 μF, in a cuvette with 0.4 cm electrode gap). G418 (200 μg/ml, Gibco) was added to the media 24 hours after plating. 8–12 days after selection, resistant ES colonies from the plates were picked using 200 μl pipette tips, dispersed with trypsin and transferred into wells of a 24 well plate. Each plate was duplicated, with one plate being frozen as a stock and the other plate used to prepare genomic DNA. G418 resistant ES clones were screened by Southern blot analysis. Genomic DNA from each clone was digested with Bam HI and 500 bp Eco RI ~ Bam HI fragment (3' to the end of homology arm) was used as a probe. Integration of the third loxP site was screened by PCR using primers flanking the two Hind III sites (which are absent when 3^rd ^loxP present) and products were digested by Hind III.

To excise the drug marker cassette (neomycin phosphotransferase and thymidine kinase), Cre recombinase was transiently expressed from pMC1-Cre plasmid, electroporated into the homologous recombinant clone cells. Gancyclovir (200 μM) was added to the media 5 days after plating. Resistant clones (those with the drug marker cassette deleted) were analysed by Southern blot using the same probe as the initial screening (see above). Cell lines were chosen in which the exon is deleted together with the drug marker cassette (*fak *LP1 allele). These were subjected to a second round of targeting, using the same targeting vector. Clones containing one knockout allele (*fak *LP1) and one allele targeted with the LP3 vector were selected. Further cre-mediated deletion of these cells generated homozyygous FAK null (LP1/LP1) ES cells, which were selected by gancyclovir resistance as before. (see Fig 1 and [9]).

### Neuronal differentiation of embryonic stem cells and electrophysiological recording

Confluent ES cells (grown as above) were trypsinised, diluted and replated and grown to confluence once more. These confluent ES cells were then treated briefly (~60 s) with trypsin, such that cells mostly detached from the flask in clumps of ~100 cells. These were then grown in suspension culture in bacterial grade Petri dishes, in GMEM/10% FCS, without LIF. Medium was changed every 48 hrs. From days 4–9, 1 μM all trans retinoic acid was added to the suspension medium. On day 9 (after '4+5' RA+/-) the embryoid bodies were collected, washed twice with phosphate buffered saline (PBS), treated with trypsin for 10 minutes and disaggregated by drawing up and down a flamed Pasteur pipette to yield a single cell suspension. This was centrifuged and the pellet resuspended in GMEM/10% FCS and cells plated at densities of 0.5 – 2.0 × 10^5 ^cells/ml on laminin coated glass coverslips. After 48 hrs the medium was replaced by Neurobasal^© ^medium supplemented with B27, 1% FCS and 0.5 mM glutamine.

Extracellular recording solution: NaCl 140, KCl 3, CaCl_2 _2.5, HEPES 15, glucose 10, MgCl_2 _0 or 1 mM, TTX 0 or 50 nM, pH 7.4 with NaOH. For calcium current recordings: NaCl 100, TEA-acetate 20, CsCl 5, KCl 3, CaCl_2 _10, HEPES 15, glucose 15, MgCl_2 _1 mM, TTX 200 nM, pH 7.4 with NaOH. Drugs/toxins were applied by bath perfusion. NMDA was microperfused via a solenoid valve operated U-tube. The composition of the recording electrode solution was (mM): K-gluconate 117.5, K-MeSO_4_, NaCl 8, HEPES 10, Mg-ATP 4, Na_2_GTP 0.3, EGTA 0.5, pH 7.25 with KOH. For calcium current recordings: CsMeSO_3 _100, CsBAPTA 5, HEPES 15, MgATP 4, NaGTP 0.4, sucrose 40; pH 7.2 with CsOH. Recording electrodes were pulled from borosilicate glass (Clark GC-150TF) on a Sutter P-87.

Currents were recorded and analysed using an Axopatch 1D amplifier and pClamp6 software (Axon Instruments).

### Immunocytochemistry and Western blotting

For immunofluorescent staining of ESN cultures, cells (grown on glass coverslips) were first fixed with cold methanol (5 mins) and washed (PBS with 5% horse serum, 0.1% Triton X-100 and 0.02% NaN_3_) and treated with blocking solution (wash solution plus 5% goat serum). Primary antibody (MAP2B; mouse monoclonal, clone 18, Transduction Laboratories or synaptophysin; mouse monoclonal, clone SY 38, Boehringer Mannheim) was then added (1:200 dilution in wash solution) and incubated for 2 hrs after which coverslips were washed 4 times and secondary antibody (Cy3 conjugated goat anti-mouse, Jackson Laboratories) was added for 1 hr. After a further 4 washes and a final rinse in distilled water, coverslips were mounted on slides using Fluoromount and viewed on an Olympus Vannox microscope. For Western blotting, cells were lysed on ice in RIPA buffer (150 mM NaCl, 1.0% NP-40, 0.5% DOC, 0.1% SDS, 50 mM Tris/Cl pH 8.0 contains 50 mM NaF, 20 μM ZnCl_2_, 1 mM Sodium orthovanadate, 0.5 mM PMSF and protease inhibitor cocktail (cϕmplete™, Boehringer Mannheim). Lysates were quantified by BCA™ Protein Assay Reagent Kit (Pierce).

Samples were run on 8% SDS polyacrylamide gel and blotted on PVDF membrane. Anti-FAK monoclonal antibody and anti-phosphotyrosine-RC20 antibody were from Transduction Laboratory. Horseradish peroxidase linked anti-mouse antibody (sheep) and ECL™ western blotting detection reagents were from Amersham Pharmacia Biotech.

Total cellular RNA was extracted from ESNs using Rneasy from Qiagen. Single strand cDNA was synthesised from this RNA using oligo(dT) primers, Superscript II and dNTP mix (Gibco). The polymerase chain reaction, using primers hybridising either side of the neuronal specific exons [22] was performed on this cDNA.

## Authors' contributions

PC carried out the electrophysiological and immunocytochemical studies and drafted the manuscript. NHK designed and constructed the targeting vector generated FAK null ES cells and performed Western blots. PC and NHK prepared ESNs by *in vitro *differentiation of ES cells. PC, NHK & SGNG conceived and designed the study, read and approved the final manuscript.

## Supplementary Material

Additional File 1 Figure showing in detail the construction of the targeting vector, together with legend. Click here for file
