# Supplementary material for: Homozygous mutation of focal adhesion kinase in embryonic stem cell derived neurons: normal electrophysiological and morphological properties in vitro
Source: BMC Neurosci. 2006 Jun 12;7:47. doi: 10.1186/1471-2202-7-47 (PMC1538614; doi:10.1186/1471-2202-7-47)
Supplement: Additional File 1 — Figure showing in detail the construction of the targeting vector, together with legend. [file 1471-2202-7-47-S1.ppt]

## Slide 1
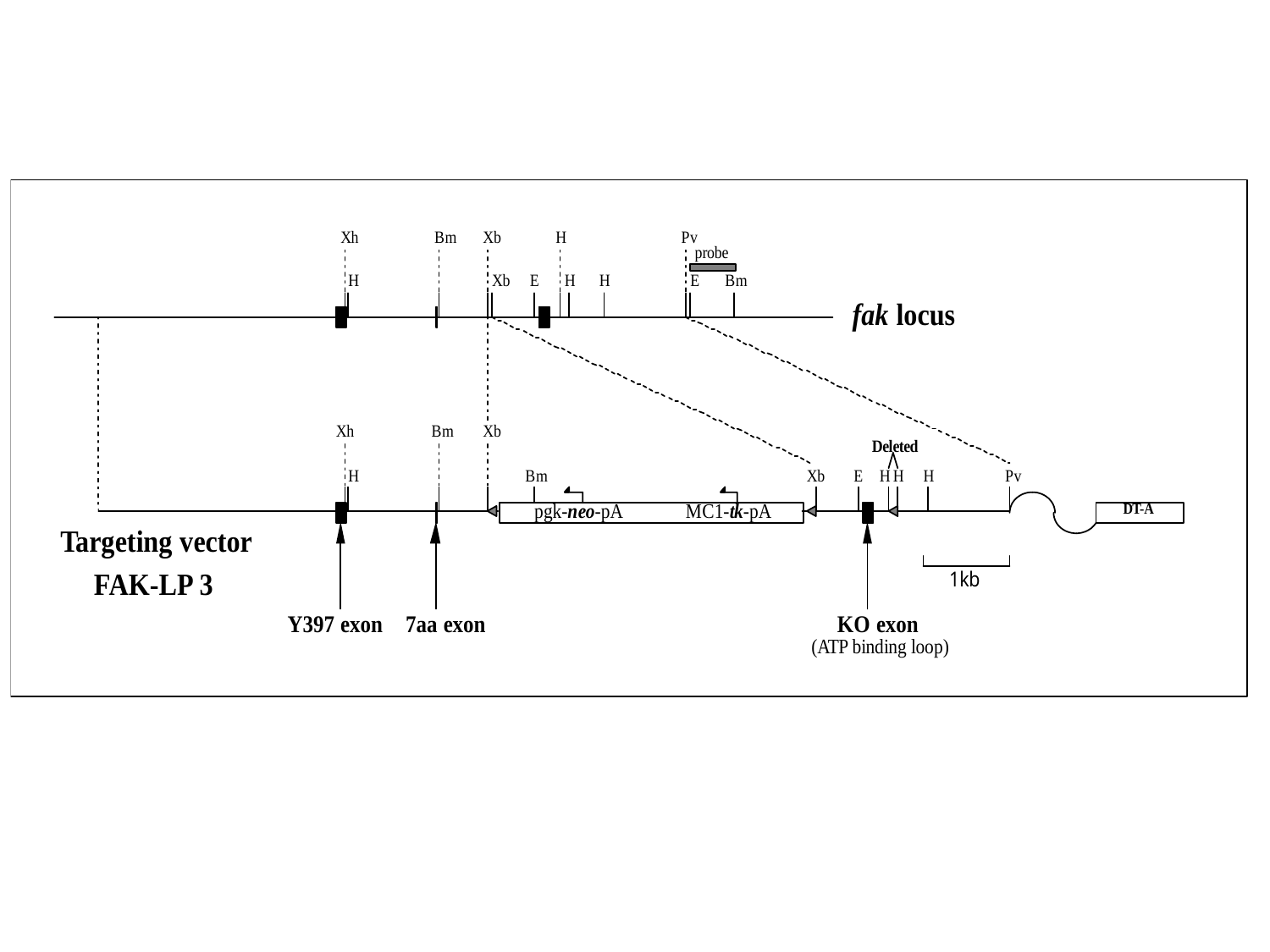

## Slide 2
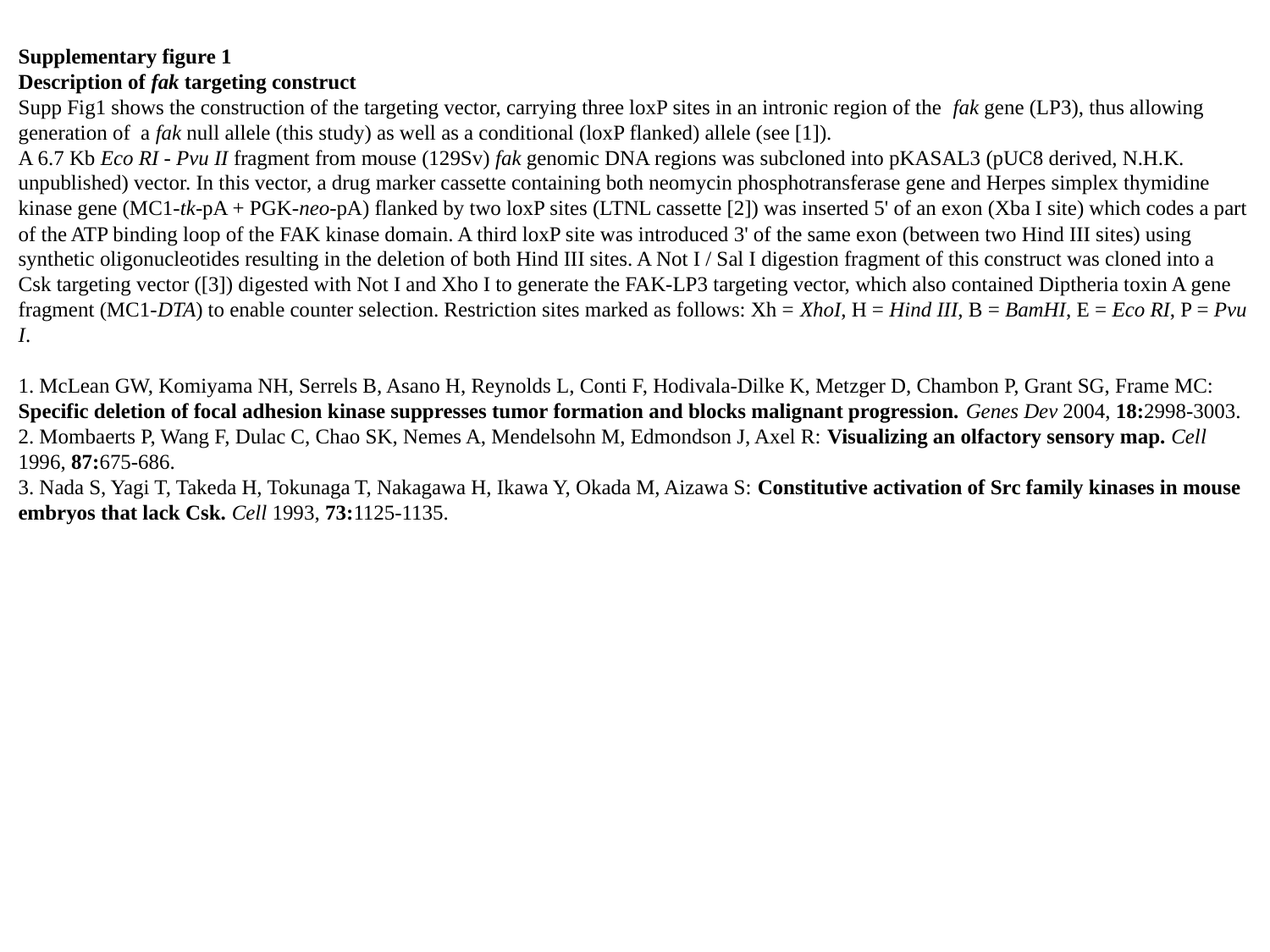

Supplementary figure 1
Description of fak targeting construct
Supp Fig1 shows the construction of the targeting vector, carrying three loxP sites in an intronic region of the fak gene (LP3), thus allowing generation of a fak null allele (this study) as well as a conditional (loxP flanked) allele (see [1]).
A 6.7 Kb Eco RI - Pvu II fragment from mouse (129Sv) fak genomic DNA regions was subcloned into pKASAL3 (pUC8 derived, N.H.K. unpublished) vector. In this vector, a drug marker cassette containing both neomycin phosphotransferase gene and Herpes simplex thymidine kinase gene (MC1-tk-pA + PGK-neo-pA) flanked by two loxP sites (LTNL cassette [2]) was inserted 5' of an exon (Xba I site) which codes a part of the ATP binding loop of the FAK kinase domain. A third loxP site was introduced 3' of the same exon (between two Hind III sites) using synthetic oligonucleotides resulting in the deletion of both Hind III sites. A Not I / Sal I digestion fragment of this construct was cloned into a Csk targeting vector ([3]) digested with Not I and Xho I to generate the FAK-LP3 targeting vector, which also contained Diptheria toxin A gene fragment (MC1-DTA) to enable counter selection. Restriction sites marked as follows: Xh = XhoI, H = Hind III, B = BamHI, E = Eco RI, P = Pvu I.
1. McLean GW, Komiyama NH, Serrels B, Asano H, Reynolds L, Conti F, Hodivala-Dilke K, Metzger D, Chambon P, Grant SG, Frame MC: Specific deletion of focal adhesion kinase suppresses tumor formation and blocks malignant progression. Genes Dev 2004, 18:2998-3003.
2. Mombaerts P, Wang F, Dulac C, Chao SK, Nemes A, Mendelsohn M, Edmondson J, Axel R: Visualizing an olfactory sensory map. Cell 1996, 87:675-686.
3. Nada S, Yagi T, Takeda H, Tokunaga T, Nakagawa H, Ikawa Y, Okada M, Aizawa S: Constitutive activation of Src family kinases in mouse embryos that lack Csk. Cell 1993, 73:1125-1135.
